# Functional G-Protein-Coupled Receptor (GPCR) Synthesis: The Pharmacological Analysis of Human Histamine H1 Receptor (HRH1) Synthesized by a Wheat Germ Cell-Free Protein Synthesis System Combined with Asolectin Glycerosomes

**DOI:** 10.3389/fphar.2018.00038

**Published:** 2018-02-06

**Authors:** Yasuyuki Suzuki, Tomio Ogasawara, Yuki Tanaka, Hiroyuki Takeda, Tatsuya Sawasaki, Masaki Mogi, Shuang Liu, Kazutaka Maeyama

**Affiliations:** ^1^Department of Pharmacology, Ehime University Graduate School of Medicine, Toon, Japan; ^2^Proteo-Science Center, Ehime University, Matsuyama, Japan; ^3^Advanced Research Support Center, Division of Analytical Bio-Medicine, Ehime University, Toon, Japan

**Keywords:** GPCRs, cell free, liposome, histamine, proteoliposome, membrane protein, glycerosome

## Abstract

G-protein-coupled receptors (GPCRs) are membrane proteins distributed on the cell surface, and they may be potential drug targets. However, synthesizing GPCRs *in vitro* can be challenging. Recently, some cell-free protein synthesis systems have been shown to produce a large amount of membrane protein combined with chemical chaperones that include liposomes and glycerol. Liposomes containing high concentrations of glycerol are known as glycerosomes, which are used in new drug delivery systems. Glycerosomes have greater morphological stability than liposomes. Proteoglycerosomes are defined as glycerosomes that contain membrane proteins. Human histamine H_1_ receptor (HRH1) is one of the most studied GPCRs. In this study, we synthesized wild-type HRH1 (WT-HRH1) proteoglycerosomes and D107A-HRH1, (in which Asp107 was replaced by Ala) in a wheat germ cell-free protein synthesis system combined with asolectin glycerosomes. The mutant HRH1 has been reported to have low affinity for the H_1_ antagonist. In this study, the amount of synthesized WT-HRH1 in one synthesis reaction was 434 ± 66.6 μg (7.75 ± 1.19 × 10^3^pmol). The specific binding of [^3^H]pyrilamine to the WT-HRH1 proteoglycerosomes became saturated as the concentration of the radioligand increased. The dissociation constant (*Kd*) and maximum density (*Bmax*) of the synthesized WT-HRH1 were 9.76 ± 1.25 nM and 21.4 ± 0.936 pmol/mg protein, respectively. However, specific binding to D107A-HRH1 was reduced compared with WT-HRH1 and the binding did not become saturated. The findings of this study highlight that HRH1 synthesized using a wheat germ cell-free protein synthesis system combined with glycerosomes has the ability to bind to H_1_ antagonists.

## Introduction

The large-scale synthesis of functional G-protein-coupled protein receptors (GPCRs), which are known to be targets of various drugs (Lagerström and Schiöth, 2008), is a challenging experimental process. Investigation of the three-dimensional (3D) structures of GPCR molecules is a major objective of research as understanding the 3D structures is an essential prerequisite for developing target-directed drugs and rationally designed screening strategies, which are key approaches used in the pharmaceutical industry (Shimamura et al., 2011; Yinfeng et al., 2016). Most structural and functional approaches to the study of GPCRs require large-scale production of the receptor protein. However, synthesizing a sufficient amount of functional GPCRs is limited by two main factors. First, classical synthesis methods, such as the overexpression of protein in cultured cells, yield GPCRs in small amounts, and overexpressed GPCRs can result in cytotoxic effects (Inglese et al., 2007). Second, GPCRs are membrane-bound proteins that favor a hydrophobic environment over a hydrophilic one, and synthesized GPCRs can aggregate rapidly in an aqueous reaction mixture.

Recently, a cell-free protein synthesis system has been found to achieve synthesis of an adequately large amount of protein (Madin et al., 2000; Katzen et al., 2009; Sonnabend et al., 2017). The eukaryotic cell-free system results in improved protein folding when compared with prokaryotic cell-free systems, such as the *E. coli* cell-free system. Also, eukaryotic cell-free systems made from rabbit reticulocytes or insect cells produce insufficient synthesized protein. However, the wheat germ system can provide a sufficiently large amount of protein (Junge et al., 2011; Harbers, 2014).

To prevent the aggregation of membrane proteins, detergents and lipid bilayer components such as liposomes and nanodiscs, which are effective in hydrophobic environments, can be added to the synthesis mixtures (Proverbio et al., 2013; Hein et al., 2014; Denisov and Sligar, 2017; Zemella et al., 2017). By convention, the addition of detergents is used to solubilize membrane proteins and prevent protein aggregation (Shimono et al., 2009). However, some detergents have been shown to inhibit protein synthesis and can affect the functions of the synthesized protein (Ishihara et al., 2005; Nozawa et al., 2007; Corin et al., 2011). To reconstitute functional membrane proteins into liposome membranes or nanodiscs, removing the detergent by dialysis or absorption methods has been considered to be essential. This process is, however, complicated and can result in the loss of functional proteins. Hence, to prevent the loss of functional proteins, detergents were not used to reconstitute membrane proteins in this study.

The addition of liposomes to the reaction mixture prevents membrane proteins from aggregating and plays a role in inserting GPCR into the liposome membranes, and in this way, liposomes can act as a ‘chaperone’ (Nomura et al., 2008; Moritani et al., 2010; Nozawa et al., 2011; Roos et al., 2013; Niwa et al., 2015). This method is straightforward and does not inhibit the synthesis of membrane protein. The wheat germ cell-free protein synthesis system, combined with the liposome chaperone method, has been shown to provide a significant amount of dopamine D1 receptor proteoliposomes, which have ligand-binding ability (Arimitsu et al., 2014; Takeda et al., 2015). However, until now, this synthesis process has not been shown to be able to synthesize other functional GPCRs, which have ligand-binding ability.

Therefore, it is important to confirm whether the method of using a wheat germ cell-free protein synthesis system combined with liposomes can provide other functional GPCRs. To achieve increased efficiency in GPCR synthesis, liposome conditions have been shown to be optimized, when compared with previous methods, by using glycerosomes (Manca et al., 2013, 2016; Taladrid et al., 2017). Glycerosomes contain a high concentration of glycerol in liposome membranes, providing a new drug delivery system, and they have a high stability and a high fluidity (Manca et al., 2013, 2016; Taladrid et al., 2017). The high stability and fluidity of glycerosomes might improve the efficiency of GPCR function in liposome membranes.

A proteoglycerosome is defined as a glycerosome that contains membrane proteins. We aimed to synthesize wild-type (WT)-human histamine H_1_ receptor (HRH1) proteoglycerosomes using a modified method. HRH1 is a class A GPCR, and it has been investigated as a therapeutic drug target, for example, for allergy disorders (Panula et al., 2015). Also, in this study, we aimed to synthesize mutant HRH1 proteoglycerosomes that have an Ala residue at position 107 instead of an Asp residue (D107A-HRH1). This mutant receptor has been reported to have low affinity for pyrilamine, which is a first-generation H_1_ antagonist (Ohta et al., 1994; Wieland et al., 1999; Bruysters et al., 2004).

In this study, we showed that a wheat germ cell-free protein synthesis system, combined with glycerosomes, can be used to produce two functional GPCRs, namely WT-HRH1 and D107A-HRH1 on a large scale. It may be possible to produce other functional GPCRs on a large scale using this synthesis method. In this study, we showed that the WT-HRH1 proteoglycerosome generated by our wheat germ cell-free synthesis method selectively bound the histamine H1 antagonists and agonist and that the specific binding of [^3^H]pyrilamine to the synthesized WT-HRH1 proteoglycerosomes became saturated as the concentration of the radioligand increased, but that this specific binding to the D107A mutant receptor proteoglycerosomes was weak, in contrast to the WT-HRH1 binding.

## Materials and Methods

### Drugs, Chemicals, and Reagents

The specific HRH1 antagonist [^3^H]pyrilamine was used in saturation and competitive binding experiments, at 20.0 Ci/mmol (Perkin-Elmer, Waltham, MA, United States), to label histamine receptors. The synthesis of protein labeled with carbon fourteen (^14^C) was produced using [^14^C]leucine (20.0 Ci/nmol) (Perkin-Elmer, Waltham, MA, United States). The following drugs were used as cold competitors: doxepin hydrochloride (Wako, Osaka, Japan), epinastine hydrochloride (Sigma–Aldrich, St. Louis, MO, United States), pyrilamine maleate (Sigma–Aldrich, St. Louis, MO, United States), ranitidine hydrochloride (Sigma–Aldrich, St. Louis, MO, United States), thioperamide (R&D Systems, Minneapolis, MN, United States), and alpha-methylhistamine (Sigma–Aldrich, St. Louis, MO, United States). Tripelennamine hydrochloride (pyribenzamine; Sigma–Aldrich, St. Louis, MO, United States) stabilized the HRH1 during the synthesis reaction.

### Glycerosome Preparation

Asolectin (Sigma–Aldrich, St. Louis, MO, United States) was dissolved in chloroform and ice-cold acetone by gently rotating at room temperature for 2 h. After standing in solution overnight at 4°C, the pellet was placed in a glass tube and gently evaporated using a stream of nitrogen gas (Nozawa et al., 2011). Evaporated asolectin was hydrated with MilliQ water containing 20% glycerol and 6 mM dithiothreitol. To completely hydrate the lipids and mix the glycerol with the liposomes, the lipid solution was placed in a sonicator bath filled with ice-cold water for 15–20 min. The final asolectin concentration of the stock solution was adjusted to 200 mg/ml. To demonstrate the size stability of the glycerosomes, asolectin liposomes containing no glycerol and 10% glycerol, respectively, were prepared by the same process. The peak diameter and polydispersity index of empty glycerosomes and proteoglycerosomes were measured using a Zetasizer Nano ZS (Malvern Instruments, Malvern, United Kingdom) after uniformalization in a sonicator bath for 1 min.

### Wheat Germ Cell-Free Protein Synthesis

Details of the wheat germ cell-free reaction have been described previously (Nozawa et al., 2011; Takeda et al., 2015). Briefly, cell-free protein synthesis was conducted using the WEPRO 7240 Expression Kit (Cell-Free Sciences, Matsuyama, Japan). WEPRO7240 is a highly concentrated wheat germ extracts. The kit included SUB-AMIX SGC, which contains 20 amino acids. The open reading frame (ORF) of the HRH1 (Uniport no. P35367) was amplified by polymerase chain reaction (PCR) using full-length cDNA clones, obtained from the Mammalian Gene Collection (MGC^1^), as the template (Strausberg et al., 1999). The amplified PCR product was subcloned into a pEU-E01-MCS vector^2^, using the Gateway system (Thermo Fisher, Waltham, MA, United States) (Takai et al., 2010), and the resultant plasmid was used as the transcription template. Mutant HRH1, in which Asp107 is replaced with Ala (D107A), was created by site-directed mutagenesis, using a Prime STAR mutagenesis basal kit (Takara, Otsu, Japan). *In vitro* transcription was performed using SP6 polymerase which was included in the WEPRO 7240 Expression Kit.

Before translation, pretreated asolectin glycerosomes, whose size was uniformed by sonicator bath briefly, were mixed with a combination of SUB-AMIX SGC and WEPRO7240 solution by gently rotating at 4°C for 1 h. The ununiformed liposomes were separated by centrifugation at 2,400 × *g* for 15 min at 4°C, and the supernatant containing uniformed liposomes was used to translate the proteins.

Translation reactions were performed using a bilayer-dialysis method as follows. First, 500 μL of the reaction mixture (125 μL WEPRO7240 wheat germ extract, 125 μL mRNA, 80 μg/mL creatine kinase, and 20 mg/ml asolectin glycerosomes) was overlaid with 2.5 mL SUB-AMIX SGC solution in a 10-K MWCO Slide-A-Lyzer dialysis device (Thermo Fisher, Waltham, MA, United States). Then, the container was immersed in 5.5 mL dialysis solution (SUB-AMIX SGC). These solutions contained 4 μM tripelennamine hydrochloride, the histamine H_1_ antagonist, which stabilized the synthesis of HRH1 (Ratnala et al., 2004). The reaction was carried out at 22°C for 24 h. To detect the synthesis of HRH1 using autoradiography, HRH1 labeled with ^14^C was produced by same synthesis method, with [^14^C]leucine in the SUB-AMIX solutions.

The tolerance of the wheat germ cell-free system to glycerosomes was estimated by synthesizing enhanced green fluorescent protein (EGFP), which was used as an index of protein synthesis ability. Fluorescent intensity of EGFP was measured using the 485 nm excitation filter with a 535 nm emission filter.

Following the synthesis of the protein, the HRH1 proteoglycerosomes were not frozen before the purification process, as the freezing-thawing process results in rupture and reconstitution of glycerosomes. Therefore, the proteoglycerosomes that contained unnecessary protein, such as a wheat germ cell extract, were separated by purification.

Purification of cell-free synthesized HRH1 proteoglycerosomes was performed as follows. HRH1 proteoglycerosomes were collected by centrifugation at 21,400 × *g* for 20 min at 4°C, and the resultant pellet was washed with 4-(2-hydroxyethyl)-1-piperazineethanesulfonic acid (HEPES) buffer (20 mM HEPES, 150 mM NaCl, 4 mM dithiothreitol, 5 μM leupeptin, KOH, at pH 7.5) in triplicate. GPCR proteoliposomes have been recommended to be collected by ultra-highspeed centrifugation (Ratnala et al., 2004). However, in this study, in the purification method, centrifuging at >50,000 × *g* was believed to be likely to result in contamination by substances included in the wheat germ cell extract. Despite this, we carried out sodium dodecyl sulfate-polyacrylamide gel electrophoresis (SDS-PAGE) of pellets that were separated by ultracentrifugation at 50,000 × *g* and 100,000 × *g* for 20 min (in addition to SDS-PAGE after centrifugation at 21,400 × *g* for 20 min). The washed pellets were stored by freezing.

Prior to the binding assay, the pellets were resuspended in 50 mM Na-K phosphate buffer (37.8 mM Na_2_HPO_4_, 12.2 mM KH_2_PO_4_, 1 mM ethylenediaminetetraacetic acid [EDTA], pH 7.4) adjusting the concentration of HRH1 to about 200 μg/ml. The resuspended HRH1 proteoglycerosome solution was uniformalized in a sonicator bath for 1 min. The prepared proteoglycerosomes were measured for glycerosome size and lipid concentration. Glycerosomes size measurements were conducted using a Zetasizer Nano ZS (Malvern Instruments, Malvern, United Kingdom). The lipid concentration was determined using a Phospholipid C-kit (Wako Pure Chemicals, Osaka, Japan).

### Sucrose Density Gradient Centrifugation

To verify the insertion of HRH1 on the glycerosome membrane, ultracentrifugation through a sucrose step-gradient was used. The density gradient ladder consisted (bottom to top) of a 45% (w/w) sucrose layer, a 20% (w/w) sucrose layer, and a 15% (w/w) sucrose layer containing 20 mM HEPES and 150 mM NaCl, in a volume ratio of 1:1:1. The proteoglycerosome suspension was carefully layered on top of the gradient. After centrifugation (18 h, 4°C, 200,000 × *g*), the proteoliposomes migrated into the middle sucrose layer (Degrip et al., 1998). The asolectin glycerosome with no integrated membrane protein was shown at a higher position in the density-gradient than the proteoglycerosomes.

To verify the distribution of HRH1, we collected 100 μL of each fraction from top to bottom of the density-gradient after separating the [^14^C]WT-HRH1 by sucrose density gradient centrifugation. Each fraction was placed on paper filters, and these filters were boiled in 10% trichloroacetic acid. The filters were washed in ethanol, and each filter was transferred into a scintillation cocktail vial, and 3 ml PICO-FLUOR PLUS liquid scintillation cocktail (Perkin-Elmer, Waltham, MA, United States) was added. The radioactivity of [^14^C]WT-HRH1 in each fraction was determined by radiation counting for 1 min in an Aloka LSC-7200 (Aloka, Tokyo, Japan) liquid scintillation counter.

### SDS-PAGE, Autoradiography, and Western Blotting

Proteins in the glycerosomes were separated by SDS-PAGE using a 12.5% polyacrylamide gel (ATTO, Tokyo, Japan) and stained with Coomassie Brilliant Blue. Prior to SDS-PAGE, the samples were not boiled at 95°C for 5 min as membrane proteins often form large insoluble aggregates when boiled, and SDS-PAGE cannot be used to separate them. The HRH1 concentration was calculated by comparison with a standard curve for bovine serum albumin (BSA), determined by densitometric analysis with ImageJ version 1.51k. The [^14^C]-HRH1 proteoglycerosomes were separated by SDS-PAGE, and this gel was dried and placed in contact with the X-ray film for 48 h to confirm the synthesis of [14C]-HRH1 by autoradiography. The film was scanned using a Typhoon 9400 scanner (GE-Health Care, Piscataway, NJ, United States). In the Western blotting study, using NuPAGE Transfer Buffer along with an XCell SureLock Mini-Cell Electrophoresis System (Thermo Fisher, Waltham, MA, United States), the resolved HRH1 was blotted onto a polyvinylidene difluoride membrane, according to the manufacturer’s protocol. The membrane was then blocked for 1 h in phosphate-buffered saline (PBS) supplemented with 5% skimmed milk powder and 0.1% Tween-20, followed by incubation with the primary HRH1 antibody, GTX102924 (GeneTex, San Antonio, TX, United States) (dilution 1:1000) at 4°C overnight, with gentle shaking in antibody incubation buffer (PBS supplemented with 1% skimmed milk powder and 0.1% Tween-20). After washing three times with the antibody incubation buffer, the secondary antibody, conjugated with horseradish peroxidase (HRP), was added to the incubation buffer at a final concentration of 1:2000, and incubated for 2 h at room temperature. After washing with buffer, the membrane was analyzed using a Western Breeze Chromogenic Western Blot Immunodetection Kit (Thermo Fisher, Waltham, MA, United States).

### Preparation of Rat Cerebral Neuronal Membranes

Male Wistar rats (300–400 g) (Japan SLC, Hamamatsu, Japan) were housed at a constant temperature of 22°C, with a humidity of 55%, on an automatically controlled 12:12 h light:dark cycle. Animal care and research protocols were in accordance with the principles and guidelines adopted by the Animal Care Committee of Ehime University and approved by the University Committee for Animal Research.

Induction of anesthesia was performed by inhalation of approximately 3% isoflurane. After the animals had been anesthetized, the cerebral tissues were quickly dissected, washed with ice-cold PBS buffer, pooled and homogenized in a 10 × volume of ice-cold PBS buffer, pH 7.4, using a Potter glass homogenizer. The homogenate was centrifuged at 2,500 × *g* for 20 min, and the resulting supernatant was centrifuged at 40,000 × *g* for 60 min. All procedures were performed at 4°C. The final pellet was resuspended in 50 mM Na-K phosphate buffer (37.8mM Na_2_HPO_4_, 12.2 mM KH_2_PO_4_, 1 mM EDTA), which was suitable for the study of HRH1 ligand binding. Total protein concentration in the rat cerebral neuronal membrane fraction was measured using a DC Protein Assay Kit (Bio-Rad Laboratories, Hercules, CA, United States).

### Kinetics and Saturation of [^3^H]Pyrilamine Binding

The specific binding of [^3^H]pyrilamine to HRH1 proteoglycerosomes and rat cerebral neuronal membranes was measured using a filtration technique. For saturation binding studies, [^3^H]pyrilamine was used at a final concentration of 2.0–32.0 nM in 50 mM Na-K phosphate buffer containing 1 mM EDTA. Each assay was performed in triplicate using 0.10 ml aliquots containing about 5 μg of HRH1 protein. Non-specific binding was measured in the presence of 1 mM unlabeled doxepin hydrochloride. After the proteoglycerosome preparations and rat cerebral neuronal membranes had been incubated at 25°C for 60 min in a shaking water bath, the reaction was terminated by placing the assay mixture tube on ice and filtering the mixture through a grade GF/B membrane filter, pretreated with 0.5% polyethyleneimine solution. The filters were washed three times with 3 ml ice-cold 50 mM Na-K phosphate buffer, then transferred into scintillation cocktail vials, and 3 ml PICO-FLUOR PLUS liquid scintillation cocktail (Perkin-Elmer, Waltham, MA, United States) was added. Bound radioactivity was measured by counting for 1 min in an LSC-7200 liquid scintillation counter (Aloka, Tokyo, Japan).

Also, in the ligand-binding assay, rapid separation of bound ligand from unbound ligand was a critical part of the method. In general, a glass filter can separate proteoglycerosomes bound with ligands from unbound ligands, but the HRH1 proteoglycerosome size was 200–300 nm, which was too small to be trapped by the GF/B grade filter. Arimitsu et al. (2014) described the separation of dopamine D1 receptor proteoliposomes using a polypropylene filter (GH Polypro, pore size 0.45 μm), which is finer than a grade GF/B filter. Unfortunately, we found that [^3^H]pyrilamine bound to polypropylene non-specifically, and so the polypropylene filter was not suitable for the binding assay used in this study. We attempted to confirm that the HRH1 proteoglycerosomes, which were processed using the same sequence as in the radioligand-binding assay, were trapped by the GF/B filter. To calculate trapping rate by GF/B filter, the filter trapping of [^14^C]WT-HRH1 proteoglycerosomes and prefiltered [^14^C]WT-HRH1 proteoglycerosome solutions was measured using a liquid scintillation counter.

### Pharmacology of [^3^H]Pyrilamine Binding

Eight different concentrations of each cold competitor were used in the competitive binding studies. Increasing concentrations of the unlabeled ligands were incubated with 20 nM [^3^H]pyrilamine and proteoglycerosome preparations or rat cerebral neuronal membranes. Following the binding reactions, the competition assays were measured by the same process using the [^3^H]pyrilamine saturation binding assay. The data are based on experiments performed in triplicate. The experimental conditions were the same as those used in the saturation studies.

### Data Analysis

The analysis of the glycerosome morphology and the amount of synthesized HRH1 proteoglycerosomes was carried out with the software package EZR, version 1.35 (Kanda, 2013). The results are expressed as means ± standard errors (SEM). Multiple comparisons of the means were used to determine the statistical significance of the differences between groups, using the Bonferroni test. Significance was assumed to be at the 0.05 level of probability (*p*).

Saturation and competition curves were fitted by non-linear regression using GraphPad Prism (GraphPad Software Inc., La Jolla, CA, United States). The individual competition curve data are expressed as the percentage decrease in specific binding of [^3^H]pyrilamine within each experiment. Inhibition constants (*Ki*) were determined using the Cheng–Prusoff equation to correct for receptor occupancy by the radioligand (Cheng and Prusoff, 1973).

Using EZR, Student’s *t*-tests were performed, assuming unequal variance, to evaluate the statistical significance of the differences in *Ki* values of ligands for synthesized and native receptors contained in rat cerebral neuronal membranes.

## Results

### Analysis of Cell-Free Expressed HRH1

#### SDS-PAGE, Autoradiography, and Western Blotting

**FIGURE 1:**
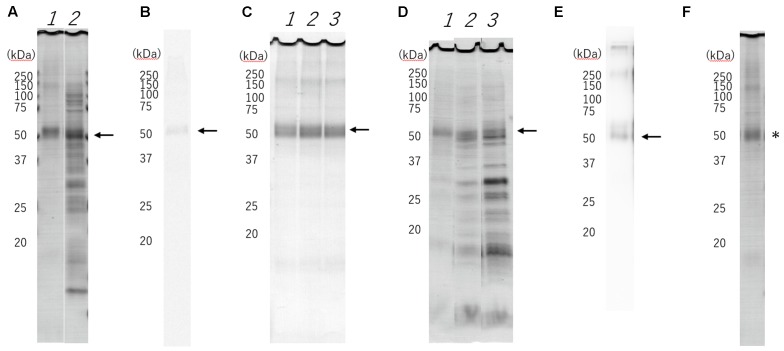
Sodium dodecyl sulfate-polyacrylamide gel electrophoresis (SDS-PAGE) separation, radioautography and Western blotting of cell-free synthesized histamine H_1_ receptor (HRH1). The expected molecular mass (56 kDa) of the HRH1 is shown (black arrow). **(A)** Purified HRH1 (lane1) by centrifugation shows the HRH1 band. Pre-purified HRH1 (lane 2) shows some contamination from the wheat germ cell-free extract. **(B)** Synthesized HRH1 in a mixture containing [14C]leucine shows a band detected by autoradiography. **(C)** HRH1 synthesized with asolectin liposome (lane 1), 10% glycerosome (lane 2), 20% glycerosome (lane 3). The glycerosomes were effective in creating conditions that allowed synthesis of HRH1 proteoglycerosomes. **(D)** Synthesized HRH1 proteoglycerosomes were purified by different centrifugation speeds. (lane 1) 21,400 × *g*, (lane 2) 50,000 × *g*, and (lane 3) 100,000 × *g* for 20 min. Centrifugation at 21,400 × *g* is suitable to purify synthesized HRH1 proteoglycerosomes. **(E)** Western blotting of synthesized HRH1 using a polyclonal antibody against HRH1 (GTX102924). **(F)** D107A-HRH1 was detected (^∗^).

We demonstrated the full scans of the entire original gels in Supplementary Figure S1. WT-HRH1 synthesized using a wheat germ cell-free protein synthesis system with asolectin glycerosomes was assessed by SDS-PAGE (lane 1 in **Figure 1A**) and autoradiography (**Figure 1B**). Western blotting indicated a band that fit the expected molecular mass (56 kDa) of WT-HRH1 (**Figure 1E**). The 20% glycerosomes were effective in creating conditions that allowed synthesis of HRH1 proteoglycerosomes (**Figure 1C** and Supplementary Figures S2–S4).

As a study had previously demonstrated that HRH1 proteoliposomes could be separated by centrifugation at 80,000 × *g* (Ratnala et al., 2004), some of the synthesized proteoglycerosomes were not separated by centrifugation at 21,400 × *g* for 20 min, and were instead separated by centrifugation at 50,000 × *g* and 100,000 × *g* for 20 min. However, we found that ultracentrifugation at >50,000 × *g* led to contamination by other substances from the wheat germ cell extract, including ribosomes and translation factors. Thus, the HRH1 proteoglycerosomes separated by centrifugation at 21,400 × *g* were purer than those separated at >50,000 × *g* (**Figure 1D**).

The amount of purified HRH1 in a single synthesis procedure was calculated based on the SDS-PAGE band intensity. The amount of WT-HRH1 proteoglycerosomes and D107A-HRH1 (**Figure 1F**) were 434 ± 66.6 μg (7.75 ± 1.19 × 10^3^ pmol) and 437 ± 68.1 μg (7.80 ± 1.22 × 10^3^ pmol), respectively. Data represent the mean ± SEM. There were no statistically significant differences in the amount of synthesized proteins (*p* = 0.969) (Student’s *t*-test) (*n* = 3).

#### Liposome Concentration

The 434 μg synthesized HRH1 proteoglycerosomes contain 1.22 mg phospholipids, which were estimated by using the Phospholipid C kit (Wako Pure Chemicals, Osaka, Japan). We determined that 4.40 mg asolectin glycerosome contained 1.22 mg phospholipid from the phospholipid assay standard curve. We therefore, determined that the HRH1/liposome ratio was approximately 1:10.

#### Liposome Size

The polydispersity index of the liposome containing 20% glycerol was significantly lower than that of the other two liposomes. This result showed that a concentration of 20% glycerol in the liposome resulted in a uniform liposome size. The Zetasizer analysis data showed that the peak diameter of HRH1 proteoglycerosomes containing 20% glycerol was 216 ± 0.318 nm after sonicating purified HRH1 proteoglycerosomes uniformly (**Table 1**). These proteoglycerosomes were classified as large unilamellar vesicles (Sharma and Sharma, 1997).

**Table 1:** Liposomes diameter measured using a Zetasizer.

Sample	Peak diameter	Polydispersity
	(nm)	index (PdI)
Empty liposome	284 ± 1.33	0.658 ± 0.0242
Empty 10% glycerosomes	136 ± 1.15	0.262 ± 0.00385
Empty 20% glycerosomes	124 ± 1.28	0.271 ± 0.00371
HRH1 proteoliposomes	334 ± 5.87	0.485 ± 0.0180
HRH1 10% proteoglycerosomes	241 ± 1.65	0.434 ± 0.00692
HRH1 20% proteoglycerosomes	216 ± 0.318	0.363 ± 0.00346


*Each peak diameter and PdI value represents the mean ± SEM (*n* = 3). The *p* values of the comparisons of the PdI of HRH1 20% proteoglycerosomes vs. HRH1 proteoliposomes and HRH1 20% proteoglycerosomes vs. 10% proteoglycerosomes were 1.0 × 10^-*6*^ and 0.0064, respectively (Bonferroni multiple comparison test).*

To confirm the stability of the diameter, we measured the sizes of the proteoliposomes and proteoglycerosomes 24 h after sonication. Prior to size measurement, proteoliposomes and proteoglycerosomes were sonicated in binding assay buffer for 10 min and the buffer containing proteoliposomes and proteoglycerosomes was incubation at 25°C for 24 h. The HRH1 proteoglycerosomes particle size slightly increased to 374.56 ± 10.65 nm. On the other hand, the HRH1 proteoliposomes particle size could not be measured as the Zetasizer alerted that HRH1 proteoliposomes were too polydisperse for cumulant analysis.

#### Sucrose Density Gradient Centrifugation

**FIGURE 2:**
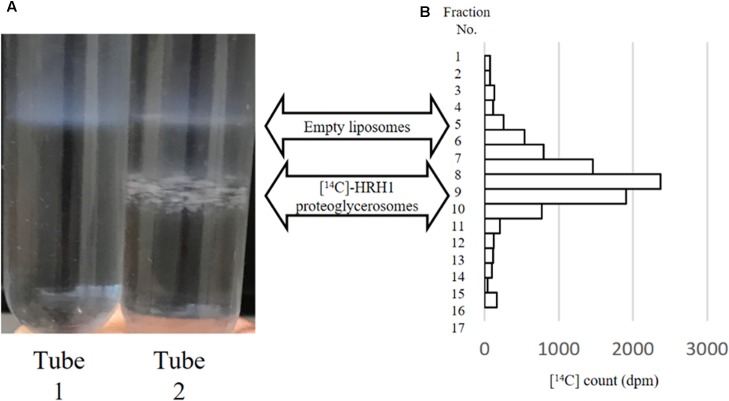
Sucrose density gradient centrifugation of empty glycerosomes and synthesized [^14^C]HRH1 proteoglycerosome. (**A**, tube 1) Following centrifugation, one turbid liposome band was detected in the mixture containing empty glycerosome. (**A**, tube 2) In contrast, two turbid liposome bands were separated in the tube containing synthesized HRH1. **(B)** [^14^C]-HRH1 radioactivity (counted using a liquid scintillation counter) of each collected fraction, from the top 100 μL to the bottom 100 μL. The highly radioactive fractions contained more synthesized [^14^C]-HRH1 proteoglycerosomes.

Sucrose density gradient centrifugation separated two different bands from the synthesized WT-HRH1 proteoglycerosomes (**Figure 2A**, tube 2). However, the empty glycerosomes showed one band after centrifugation (**Figure 2A**, tube1). Radioactivity measurements of [^14^C]-HRH1 by liquid scintillation confirmed that synthesized HRH1 was distributed in a higher density band only. The glycerosomes distributed in the low-density layer contained no synthesized protein. Also, no detection of [^14^C]-HRH1 in the lowest fraction of the sucrose density gradient showed that synthesized HRH1 was not aggregated (**Figure 2B**). This result confirmed that the glycerosome ‘chaperone’ method used in this study synthesized HRH1 by integrating into the glycerosome membranes, because proteoglycerosomes with integrated GPCR were distributed at a higher density compared with the glycerosome with no integrated membrane protein. In addition, the turbid liposome band containing HRH1, which appeared in the HRH1 proteoglycerosomes tube, is more concentrated than the band in the HRH1 proteoliposomes tube (Supplementary Figure S4). This result was critical because the integration into the membrane was necessary for the GPCRs to gain pharmacological function.

#### The Tolerance of Wheat Germ Cell-Free System to Glycerosomes

The reaction mixture containing glycerosomes produced 25% less EGFP in comparison to the EGFP only reaction mixture which contained no glycerol (Supplementary Figure S5). The high glycerol concentration slightly inhibited the synthesis of non-membrane proteins.

### Filter Grade GF/B Trapping of HRH1 Proteoglycerosomes

After filtering the [^14^C]-HRH1, which was resuspended in the same condition as in the radioligand-binding assay, using a GF/B filter, the filter was washed three times with 3 ml buffer. Comparing the ^14^C count associated with GF/B-filter-trapped [^14^C]-HRH1 with that of the pre-filtering solution showed that the GF/B filter could trap 89.8% of the HRH1 proteoglycerosomes synthesized by our method. The GF/B filter was suitable for separating the ligands binding to HRH1 from the non-bound ligands.

### Kinetics and Saturation of [^3^H]Pyrilamine Binding

**FIGURE 3:**
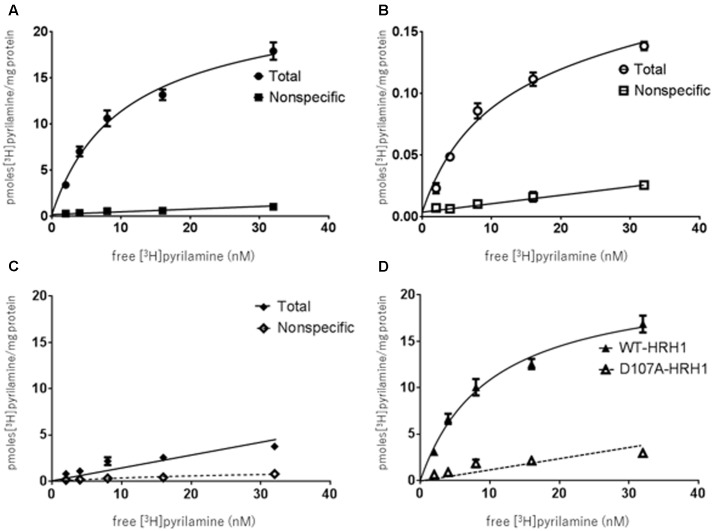
Binding characteristics of [^3^H]pyrilamine to receptor protein synthesized using the cell-free protein synthesis system and rat cerebral neuronal membranes. **(A)** [^3^H]pyrilamine saturation curve to synthesized WT-HRH1. **(B)** Saturation curve for the rat cerebral neuronal membranes. **(C)** Saturation curve for synthesized D107A-HRH1. Specific binding was calculated as the difference between total binding and non-specific binding obtained in the absence and presence of 1 mM unlabeled doxepin hydrochloride, respectively. **(D)** Specific binding of [^3^H]pyrilamine to the synthesized WT-HRH1 and D107A-HRH1 was compared. Each point represents the mean ± SEM of three experiments.

The specific binding of [^3^H]pyrilamine to the synthesized WT-HRH1 proteoglycerosomes, D107A-HRH1 proteoglycerosomes, and rat cerebral neuronal membranes was investigated. The specific binding of [^3^H]pyrilamine to the WT-HRH1 proteoglycerosomes and rat cerebral neuronal membranes became saturated as the concentration of the radioligand increased. The apparent dissociation constant (*Kd*) and the maximum number of binding sites (*Bmax*) were calculated by Prism analysis. The *Kd* and *Bmax* of the synthesized WT-HRH1 were 9.62 ± 1.25 nM and 21.4 ± 0.936 pmol/mg protein, respectively. The corresponding values for rat cerebral neuronal membranes were 9.76 ± 2.04 nM and 0.151 ± 0.0128 pmol/mg protein (**Figures 3A,B**). There was no statistical difference in *Kd* (*p* = 0.983) (Student’s *t*-test) (*n* = 3). However, specific binding to D107A-HRH1 was lower than that for WT-HRH1 and the binding did not become saturated.

### Pharmacological Study of [^3^H]Pyrilamine Binding

**FIGURE 4:**
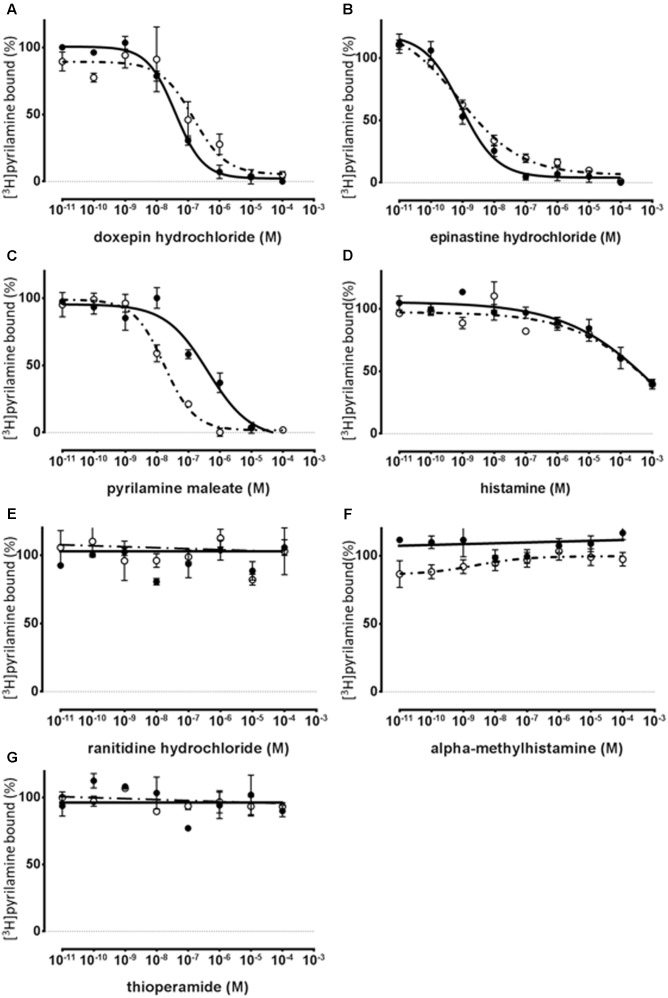
Pharmacological specificity of [^3^H]pyrilamine binding to the synthesized WT-HRH1 with the cell-free protein synthesis system and rat cerebral neuronal membranes. Competitive inhibition curves of [^3^H]pyrilamine to the synthesized receptor (•) and the rat cerebral neuronal membranes (∘). Competition binding assay for WT-HRH1 by **(A–C)** histamine H1 antagonists, **(D)** histamine H_1_ agonist, **(E)** histamine H_2_ antagonist, **(F)** histamine H_3_ agonist, and **(G)** histamine H_3_ and H_4_ antagonist. Apparent IC_50_ values were determined in competition assays with 20 nM [^3^H]pyrilamine preincubated with increasing concentrations of antagonists and an agonist (**Table 2**). Each point presents the mean ± SEM of three experiments.

To show the profile of the synthesized HRH1 proteoglycerosomes, a series of displacement-binding experiments were undertaken using a fixed concentration (20 nM) of [^3^H]pyrilamine and increasing concentrations of a histamine H_1_ antagonist or agonist. Histamine H_1_ antagonists, pyrilamine maleate, doxepin hydrochloride, and epinastine hydrochloride, produced concentration-dependent inhibition of the specific binding of [^3^H]pyrilamine to histamine H_1_ binding sites in the synthesized receptors and rat cerebral neuronal membranes (**Figures 4A–C**).

The histamine H_2_ antagonist, ranitidine hydrochloride (**Figure 4E**), and the histamine H_3_ and H_4_ antagonist, thioperamide, exhibited no displacement ability regarding [^3^H]pyrilamine binding to the synthesized receptors and rat cerebral neuronal membranes (**Figure 4G**). In the displacement-binding experiments, with a histamine H_1_ agonist, the findings showed that the *Ki* values of histamine were 0.292 mM ± 0.229 mM for the synthesized WT-HRH1 and 0.129 mM ± 0.0542 mM for the rat cerebral neuronal membranes (**Figure 4D**). The histamine H_3_ agonist, alpha methylhistamine, did not inhibit the binding ability of [^3^H]pyrilamine to the synthesized HRH1 and rat cerebral neuronal membranes (**Figure 4F**). There were no significant differences in the *Ki* values of all the H_1_ antagonists and agonist between those associated with the synthesized HRH1 proteoglycerosomes and those associated with the rat cerebral neuronal membranes (**Table 2**).

**Table 2:** The ability of ligands to displace [^3^H]pyrilamine binding to synthesized HRH1 proteoglycerosomes and rat cerebral neuronal membranes.

Competitor	Synthesized	Rat cerebral	*p*-values
	HRH1 *Ki* (nM)	neuronal membranes	
		*Ki* (nM)	
**Antagonist**			
Doxepin	13.4 ± 2.72	85.0 ± 45.0	0.189
Epinastine	0.332 ± 0.0989	0.288 ± 0.0615	0.728
Pyrilamine	181 ± 89.0	5.89 ± 1.88	0.120
**Agonist**			
Histamine	2.92 × 10^5^ ± 2.29 × 10^5^	1.29 × 10^5^ ± 0.542 × 10^5^	0.623


*Data represent the mean ± SEM, The *p*-values were determined by Student’s *t*-tests (*n* = 3).*

## Discussion

The most notable outcome of this study was the large-scale synthesis of functional WT-HRH1 and D107A HRH1 by the wheat germ cell-free protein synthesis system combined with glycerosomes. The ligand-binding ability of synthesized WT-HRH1 was greater than that of synthesized D107A-HRH1 as shown in previous studies (Ohta et al., 1994; Wieland et al., 1999; Bruysters et al., 2004).

### HRH1

Histamine is a biogenic amine and key mediator in various physiological and pathophysiological conditions, such as allergy and inflammation (Panula et al., 2015). Histamine activates four distinct histamine receptors (H_1_, H_2_, H_3_, and H_4_) that belong to the GPCR superfamily (Shahid et al., 2009). Activated HRH1 promotes several allergic reactions, and multiple H_1_ antagonists are widely prescribed to control allergic symptoms. X-ray crystallography has shown that the structure of the HRH1 complex can be modified by doxepin (Shimamura et al., 2011). HRH1 X-ray structural studies have provided information to support future studies of new drugs using virtual screening (Yinfeng et al., 2016). In this respects, HRH1 is one of the most well-studied GPCRs, and it is likely that the wheat germ cell-free protein synthesis (with glycerosomes) of HRH1 may be developed as a useful tool for the synthesis of functional GPCRs.

However, in previous structural research, the intracellular long-loop domain of the GPCRs was changed to T4L, which is a shorter domain, to stabilize the receptor structure (Shimamura et al., 2011). This modification often stabilized the synthesis of other GPCRs in cell-free systems (Yang et al., 2011). The findings of the present study have shown that a wheat germ cell-free system provides the potential for large-scale synthesis of WT-HRH1 that has ligand binding ability. Large-scale synthesis of wild-type GPCRs by the method described in this study is likely to be a useful tool for investigating the interaction between ligand binding and intracellular signal pathways because the intracellular domain of the WT-GPCR was not modified.

### Cell-Free Protein Synthesis

Cell-free protein synthesis systems produce a significant amount of proteins and are relatively easy methods. Former protein synthesis systems that used *E. coli* cell extracts achieved a significant amount of protein synthesis within several hours (Shimono et al., 2009). However, rapid transcription can result in protein misfolding and aggregation, whereas the transcription speed of the eukaryotic system is moderate, which prevents the human protein from misfolding. The current widely used protein synthesis systems involve rabbit reticulocytes, insect cells, and wheat germ. The yield of protein synthesis by wheat germ cell-free systems is the greatest out of these three systems (Harbers, 2014). The amount of HRH1, synthesized from 125 μL of WEPRO7240 wheat germ cell extracts in 24 h, is about 400 μg which is significant for the study of GPCR functions. Therefore, the wheat germ cell-free protein synthesis system may be increasingly used to synthesize human proteins.

### Functional GPCR Synthesis by Cell-Free Protein Synthesis

G-protein-coupled receptors are known as membrane proteins. Membrane proteins synthesized in an aqueous environment aggregate readily because membrane proteins have long hydrophobic domains. Furthermore, if synthesized GPCRs are folded incorrectly in the cell membrane, the function of the GPCRs may be undetectable. Therefore, there have been few studies that have demonstrated the ligand-binding ability of GPCRs synthesized by a cell-free protein synthesis system (Ishihara et al., 2005; Sansuk et al., 2008; Corin et al., 2011; Klammt et al., 2011; Yang et al., 2011; Arimitsu et al., 2014). To reduce the risk of aggregation and loss of function, a modified protein synthesis process is needed.

In previous studies, various detergents were mixed with the protein synthesis mixture to prevent the proteins from aggregating. The solubilized proteins were then reconstituted into liposome membranes by removing the detergents, which was generally carried out using dialysis or absorbance beads (Klammt et al., 2007; Shimono et al., 2009; Jørgensen et al., 2017). Sansuk et al. (2008) synthesized functional WT-HRH1 using *E. coli* cell-free systems, and according to their synthesis method, His-tagged WT-HRH1 was solubilized by detergent, solubilized WT-HRH1 was purified using Ni-nitrilotriacetic acid (NTA), and after purification, WT-HRH1 was reconstituted into asolectin liposome membranes by removing the detergent. This previous study showed that the synthesized WT-HRH1 was functional (based on a competitive analysis with various histamine H_1_ antagonists), but the study did not show that the specific binding of the synthesized WT-HRH1 became saturated, and the number of binding sites (*Bmax*) was unclear (Sansuk et al., 2008). The complicated synthesis processes and rapid synthesis by *E. coli* cell-free systems might lead to loss of functional HRH1. However, in the present study, the specific binding of the synthesized HRH1 became saturated, because our wheat germ cell-free synthesis method was a straightforward process that involved directly integrating the GPCR into the liposome membranes during protein synthesis.

In this study, we regarded the use of the chemical ‘chaperones,’ glycerol and liposome, as important because they promoted correct folding of the membrane proteins and insertion into the membranes (Figler et al., 2000; Niwa et al., 2015; Taladrid et al., 2017). The protein synthesis reaction mixture containing the chemical chaperone facilitated membrane protein folding into the lipid bilayer without requiring solubilization and reconstitution.

Previous studies have investigated the addition of asolectin liposomes into the synthesis mixture. Takeda et al. (2015) showed that dopamine D_1_ receptors, which were synthesized using a wheat germ cell-free system combined with asolectin liposomes, were integrated into the liposome membrane and bound specifically to dopamine ligands, based on the results of a BIACORE assay (Takeda et al., 2015). Arimitsu et al. (2014) showed similar findings for the dopamine D_1_ receptor, which was synthesized by the same methods, and studied using a radioisotopic ligand-binding assay, but the amount of functional dopamine D_1_ receptor was 0.02% of the total synthesized D1 receptor. Therefore, the aim of this study was to try to achieve highly efficient synthesis of functional GPCRs by improving direct integration of GPCRs into the liposome membrane. In this study, this aim was achieved in the following way.

In resuspending the asolectin liposome into stock solution, 20% glycerol was mixed completely with asolectin liposome by sonication. Liposome combined with highly concentrated glycerol, known as glycerosome, was used as the drug delivery system. The glycerosome membrane has high fluidity and stability (Manca et al., 2016). High fluidity and stability might stabilize GPCR function, as glycerol induced a dose-dependent increase in size stability of the HRH1 proteoglycerosomes (**Table 1**). The 20% glycerosomes promoted the integration of HRH1 into the membrane more effectively than standard liposomes (**Figure 1C** and Supplementary Figures S2–S4). In our pilot study, the WT-HRH1 proteoliposomes, which contained no glycerol, showed unstable affinity to histamine H1 ligands and produced large error bars (Supplementary Figure S2).

The HRH1 proteoglycerosome synthesized by our synthesis method was evaluated qualitatively. Radioactivity counts of [^14^C]WT-HRH1-containing fractions that were separated by sucrose density gradient centrifugation showed that HRH1 integrated into glycerosome membranes were distributed at the high-density level, while the glycerosomes containing no membrane protein were distributed at the low-density level (**Figure 2**). These results confirmed that our synthesis method could integrate HRH1 into glycerosome membranes during protein synthesis. Also, the synthesized HRH1 formed no protein aggregates, as demonstrated by the lack of ^14^C counts for the lowest fraction of the sucrose density gradient. If the HRH1 aggregated during the synthesis process, the aggregated HRH1 would have been distributed in the lowest fraction of the sucrose density gradient after centrifugation.

Also, in this study, the high-efficiency GPCR synthesis process produced functional HRH1, which had a high affinity for histamine H_1_ ligands. The *Kd* value of the synthesized WT-HRH1 was the same as that of the rat cerebral neuronal membranes. The specific binding of the synthesized WT-HRH1 showed that *Bmax* was 21.4 ± 0.936 pmol/mg protein, which means that 0.1% of the synthesized WT-HRH1 bound to [^3^H]pyrilamine, and the functional GPCR ratio was five times greater than the ratio reported in a previous study (Arimitsu et al., 2014). Furthermore, we showed that D107A-HRH1, which has a low affinity for the histamine H_1_ antagonist, could be synthesized by our method (**Figures 3C,D**). It should be noted that this synthesis method could potentially be used to study various mutagenesis proteins of GPCRs. Competitive examination with non-radioisotopic-labeled antagonists and agonists demonstrated that synthesized HRH1 could bind specifically to histamine H_1_ ligands. Comparison of the *Ki* values of the antagonists and the agonist indicated that there were no significant differences between the synthesized HRH1 proteoglycerosome and rat cerebral neuronal membranes (**Figure 4** and **Table 2**), and our synthesis method could provide the same experimental environment for the investigation of drugs *in vivo*. Therefore, this GPCR synthesis method may be developed as a key tool for drug discovery involving GPCRs.

### Further Direction

In previous studies, the ratio of functional GPCRs synthesized by the cell-free system was very low; 0.02% of dopamine D1 receptors (Arimitsu et al., 2014), 0.05% of β2-adrenergic receptors (Yang et al., 2011), 0.01% of endothelin B receptors (Proverbio et al., 2013), and 0.40% of endothelin B receptors have receptor functions (Zemella et al., 2017). In our study, about 0.1% of the HRH1 synthesized by our method had binding ability. Our synthesis method allowed efficient synthesis of functional GPCRs. These findings should be supported by further studies.

The first suggestion for future studies would be to investigate the ratio of protein to liposome. In the synthesis system used in this study, the weight ratio of GPCR protein to lipid was 1:10. This high density of GPCR in the liposome membrane is suitable for protein structure analysis. However, this ratio may reflect an imbalance in comparison to an *in vivo* cell membrane environment, in which there is a smaller amount of HRH1 proteins. Previously, the optimal weight ratio of GPCR to lipid membrane was shown to be 1:200–300 in a functional assay of GPCRs (Mouro-Chanteloup et al., 2010). The excessive density of single GPCR in a glycerosome membrane might inhibit the GPCR functions, and optimizing this ratio could improve the efficiency of functional GPCR synthesis using our method. To optimize this ratio, adding a greater quantity of glycerosomes into the reaction mixture might be effective. However, excessive glycerosomes might inhibit the synthesis reaction. In future studies, the reconstitution of HRH1 proteoglycerosomes into additional glycerosomes, adjusted to the optimal protein/lipid ratio after HRH1 synthesis, should be explored using the wheat germ cell-free protein synthesis method.

The second future area of study would involve inserting GPCR into the liposome membrane. Previous studies have shown that synthesized membrane protein was reconstituted by unidirectional insertion (Knol et al., 1996; Roos et al., 2013). However, other studies have shown that the ratio of forward GPCR insertion to inverse insertion was random (Smith and Morrissey, 2004; Takeda et al., 2015). Unfortunately, our method could not control the direction of insertion of GPCRs into the liposome membrane. Therefore, the ratio of functional GPCRs to total synthesized GPCRs might have been underestimated in our study. We recommend that future studies should include an exploration of unidirectional insertion of GPCRs into the membrane.

The third area to be studied was the modification of the lipid bilayer environment. Recent studies have demonstrated that the nanodisc was more uniform and stable in comparison to the liposomes. Due to these advantages, the nanodisc was more preferable for reconstituting GPCRs into the lipid bilayer membrane (Proverbio et al., 2013; Hein et al., 2014; Denisov and Sligar, 2017; Zemella et al., 2017). Furthermore, specific phosphatidylglycerols such as dioleoylphosphatidylglycerol (DOPG) have been shown to have high affinity to GPCRs (Inagaki et al., 2012; Dawaliby et al., 2016). Hence, the lipid bilayer containing highly concentrated DOPG could potentially be a suitable membrane to fold the GPCR structure. Future studies to incorporate GPCRs into the nanodisc or lipid bilayer containing DOPG could thus potentially demonstrate high ligand binding ability.

A fourth point that should be investigated in future studies is the difference in the affinity for pyrilamine between synthesized HRH1 and rat cerebral neuronal membranes. However, there appeared to be no significant statistical difference in our study. Sansuk et al. (2008) showed that HRH1 synthesized by an *E. coli* cell-free protein synthesis system indicated similar findings. Previous research on GPCRs synthesized without G-proteins indicated that the GPCRs had low affinity for some antagonists and agonists because they were not in their active state (Fitzsimons et al., 2004). However, homogenates of HEK293T cells transiently expressing the HRH1 demonstrated that the affinity for pyrilamine was comparable to that described in previous studies (Bosma et al., 2017) because this homogenate included G-proteins. To achieve a synthesis of active state GPCRs, which have the same binding affinity for all ligands, a future study of the co-synthesis of HRH1 and G-proteins is suggested. To carry out this study, focusing on the interaction between GPCRs and G-proteins will be important. Our synthesis method has the benefit of making this interaction possible because our method allows the synthesis of functional WT-GPCRs with non-modified intracellular domains.

## Conclusion

We have demonstrated, for the first time, that a large quantity of functional GPCRs were synthesized by a wheat germ cell-free system combined with glycerosomes. We are confident that this method will become a key tool in the investigation of the function of GPCRs and the discovery of new drugs that target orphan GPCRs.

## Author Contributions

YS performed the research and wrote the manuscript. YT constructed vectors. TO, HT, and TS synthesized the protein by cell-free systems. SL, MM, and KM planned and supervised the writing of the manuscript.

## Conflict of Interest Statement

The authors declare that the research was conducted in the absence of any commercial or financial relationships that could be construed as a potential conflict of interest.

## Footnotes

1 https://genecollections.nci.nih.gov/MGC/

2 http://www.cfsciences.com/documents/pEU-E01-MCS_v2.pdf
