# Supplementary material for: Functional G-Protein-Coupled Receptor (GPCR) Synthesis: The Pharmacological Analysis of Human Histamine H1 Receptor (HRH1) Synthesized by a Wheat Germ Cell-Free Protein Synthesis System Combined with Asolectin Glycerosomes
Source: Front Pharmacol. 2018 Feb 6;9:38. doi: 10.3389/fphar.2018.00038 (PMC5808195; doi:10.3389/fphar.2018.00038)
Supplement: Supplementary file 1 [file Presentation_1.pptx]

## Slide 1
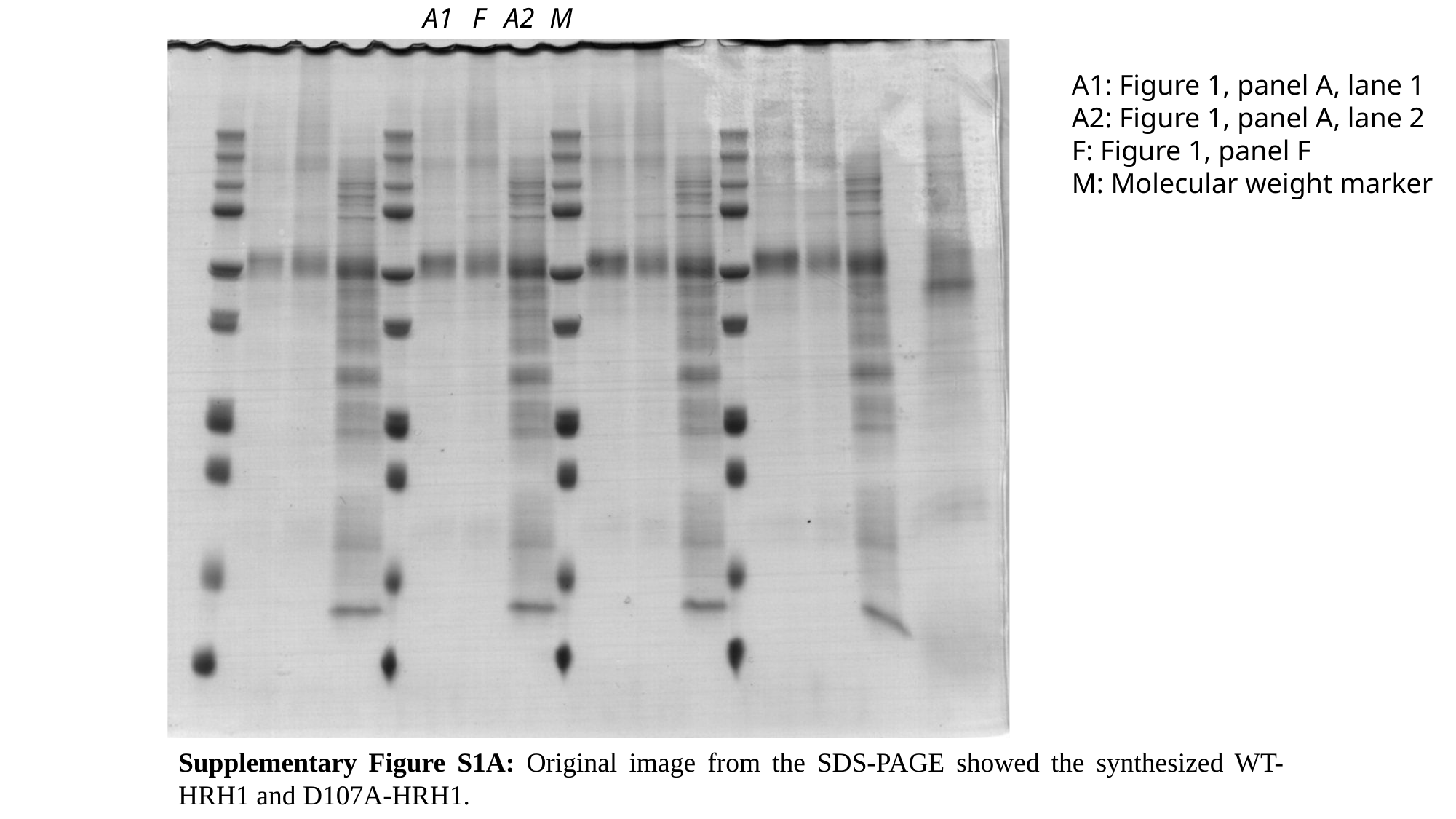

A1
F
A2
M
A1: Figure 1, panel A, lane 1
A2: Figure 1, panel A, lane 2
F: Figure 1, panel F
M: Molecular weight marker
Supplementary Figure S1A: Original image from the SDS-PAGE showed the synthesized WT-HRH1 and D107A-HRH1.

## Slide 2
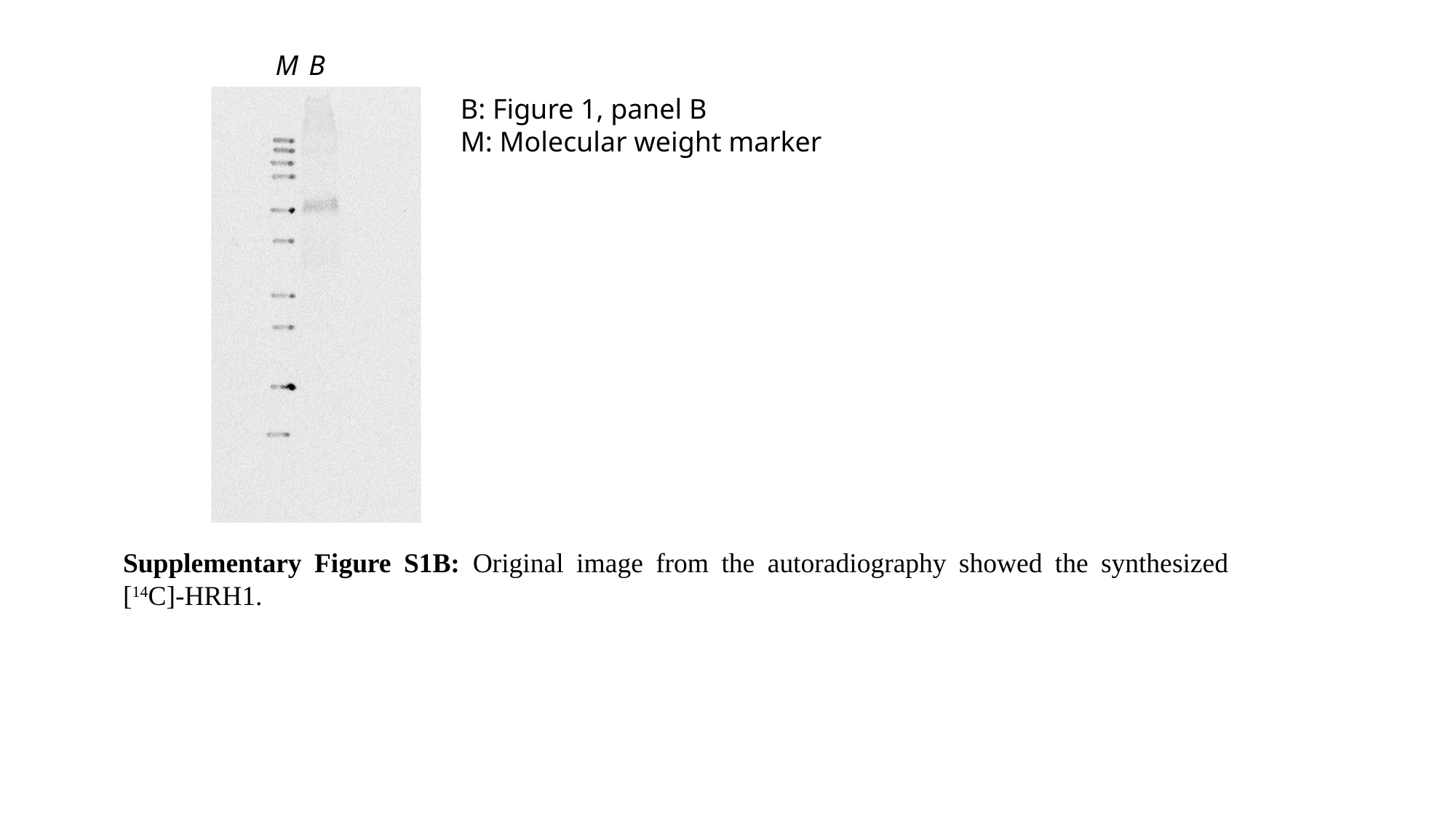

M
B
B: Figure 1, panel B
M: Molecular weight marker
Supplementary Figure S1B: Original image from the autoradiography showed the synthesized [14C]-HRH1.

## Slide 3
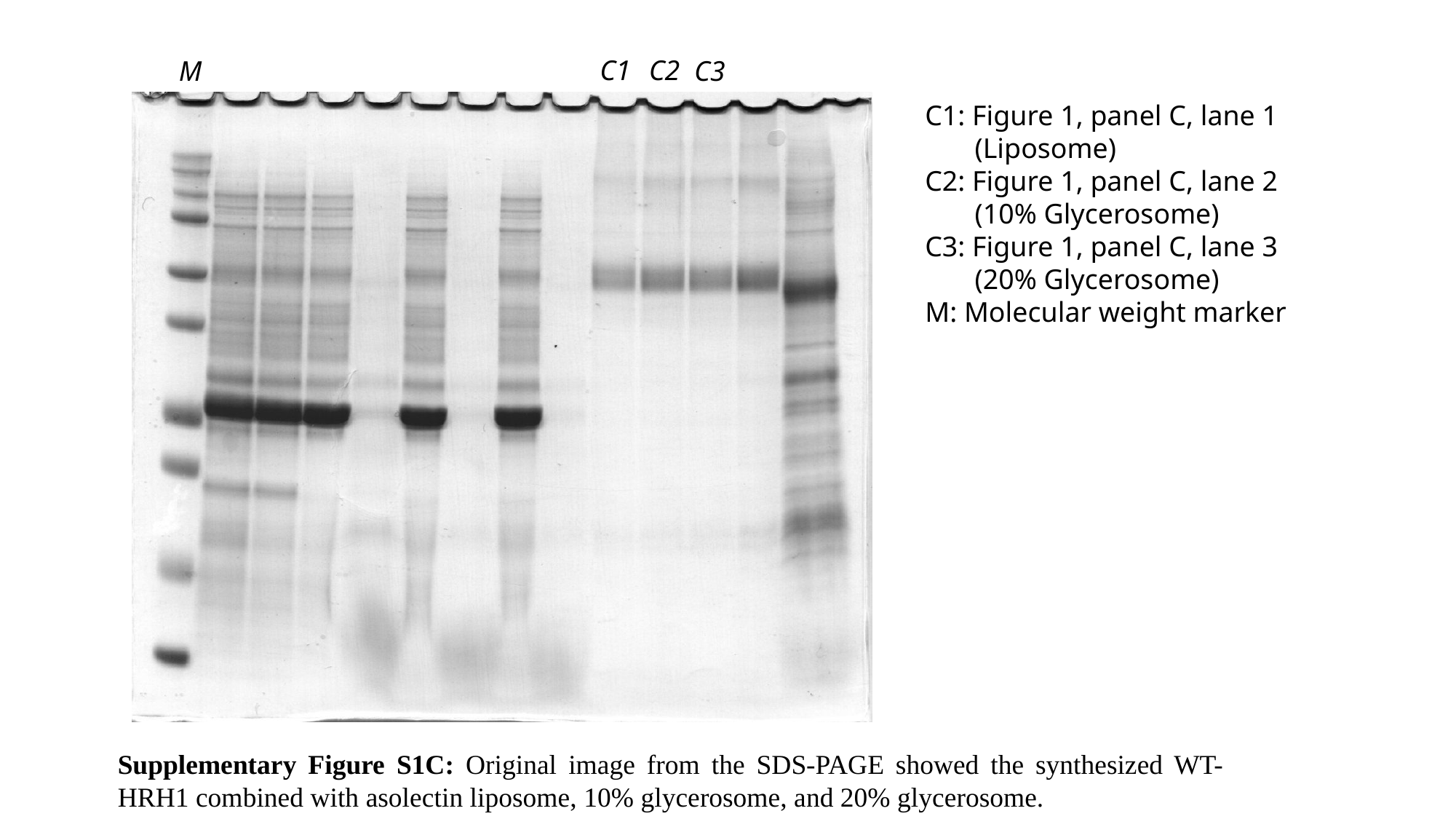

C1
C2
M
C3
C1: Figure 1, panel C, lane 1
 (Liposome)
C2: Figure 1, panel C, lane 2
 (10% Glycerosome)
C3: Figure 1, panel C, lane 3
 (20% Glycerosome)
M: Molecular weight marker
Supplementary Figure S1C: Original image from the SDS-PAGE showed the synthesized WT-HRH1 combined with asolectin liposome, 10% glycerosome, and 20% glycerosome.

## Slide 4
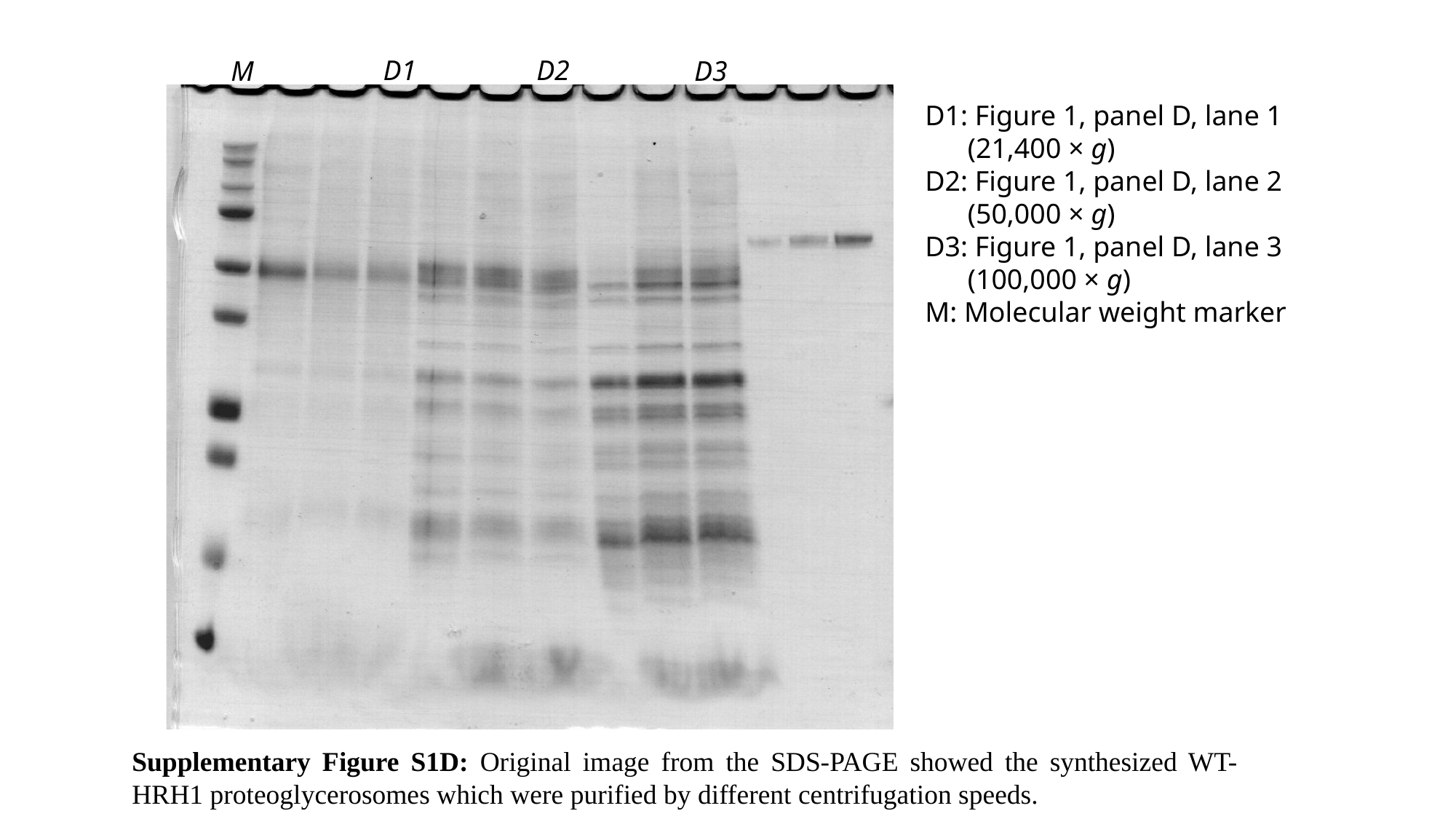

D1
D2
M
D3
D1: Figure 1, panel D, lane 1
 (21,400 × g)
D2: Figure 1, panel D, lane 2
 (50,000 × g)
D3: Figure 1, panel D, lane 3
 (100,000 × g)
M: Molecular weight marker
Supplementary Figure S1D: Original image from the SDS-PAGE showed the synthesized WT-HRH1 proteoglycerosomes which were purified by different centrifugation speeds.

## Slide 5
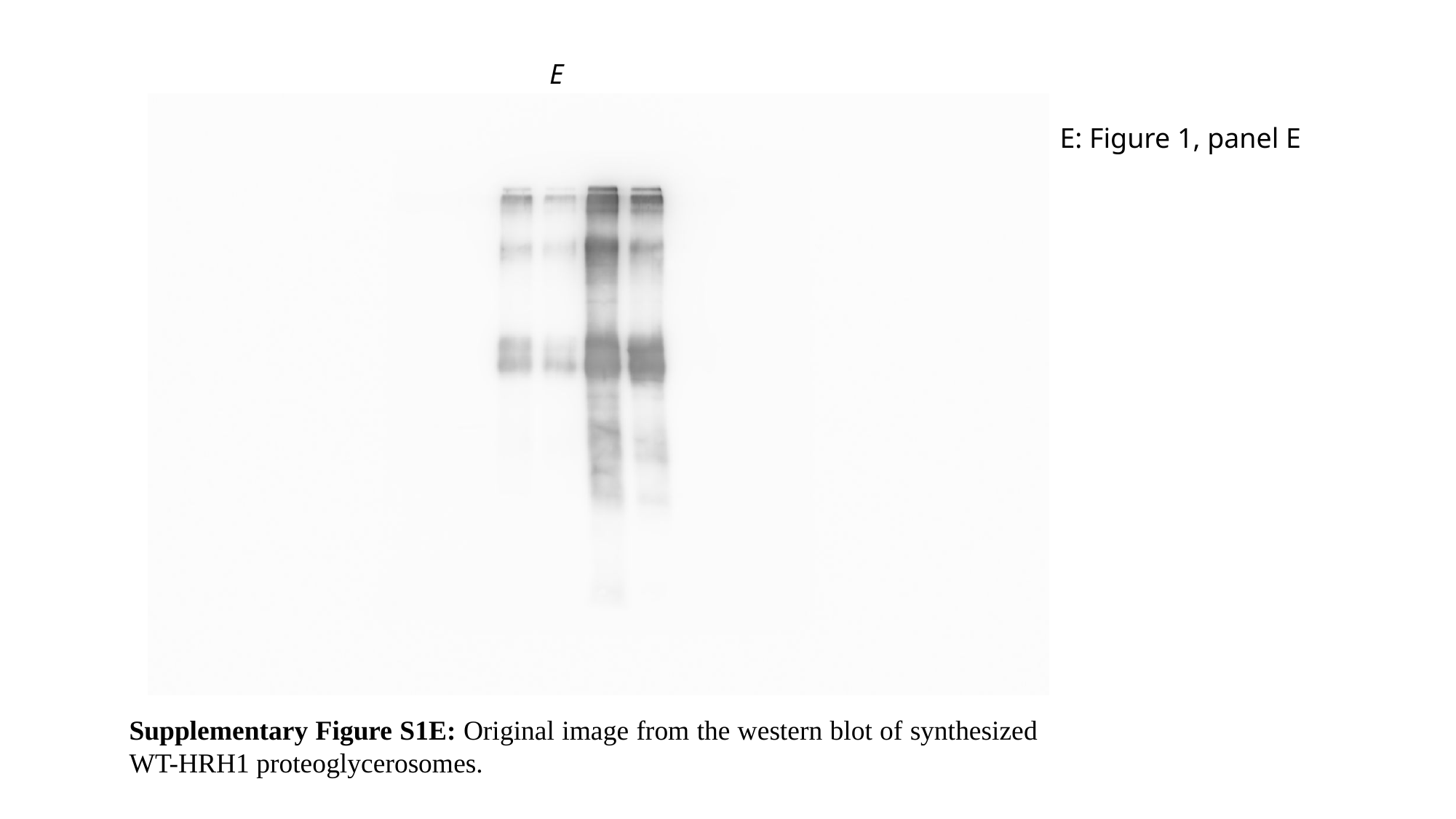

E
E: Figure 1, panel E
Supplementary Figure S1E: Original image from the western blot of synthesized WT-HRH1 proteoglycerosomes.
