# Supplementary material for: Functional G-Protein-Coupled Receptor (GPCR) Synthesis: The Pharmacological Analysis of Human Histamine H1 Receptor (HRH1) Synthesized by a Wheat Germ Cell-Free Protein Synthesis System Combined with Asolectin Glycerosomes
Source: Front Pharmacol. 2018 Feb 6;9:38. doi: 10.3389/fphar.2018.00038 (PMC5808195; doi:10.3389/fphar.2018.00038)
Supplement: Supplementary file 2 [file Presentation_2.pptx]

## Slide 1
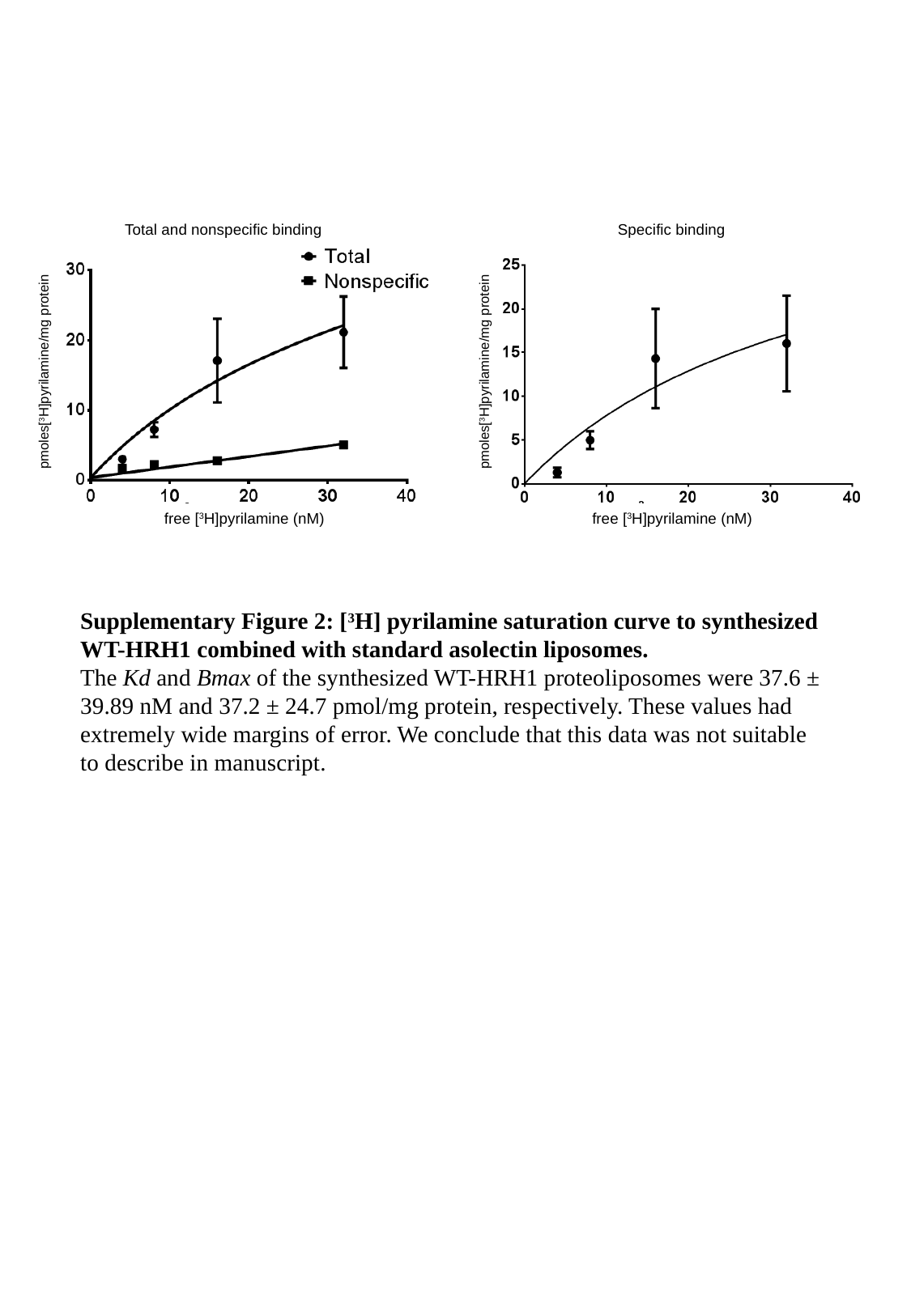

Total and nonspecific binding
Specific binding
pmoles[3H]pyrilamine/mg protein
pmoles[3H]pyrilamine/mg protein
free [3H]pyrilamine (nM)
free [3H]pyrilamine (nM)
Supplementary Figure 2: [3H] pyrilamine saturation curve to synthesized WT-HRH1 combined with standard asolectin liposomes.
The Kd and Bmax of the synthesized WT-HRH1 proteoliposomes were 37.6 ± 39.89 nM and 37.2 ± 24.7 pmol/mg protein, respectively. These values had extremely wide margins of error. We conclude that this data was not suitable to describe in manuscript.
