# Supplementary material for: Functional G-Protein-Coupled Receptor (GPCR) Synthesis: The Pharmacological Analysis of Human Histamine H1 Receptor (HRH1) Synthesized by a Wheat Germ Cell-Free Protein Synthesis System Combined with Asolectin Glycerosomes
Source: Front Pharmacol. 2018 Feb 6;9:38. doi: 10.3389/fphar.2018.00038 (PMC5808195; doi:10.3389/fphar.2018.00038)
Supplement: Supplementary file 3 [file Presentation_3.pptx]

## Slide 1
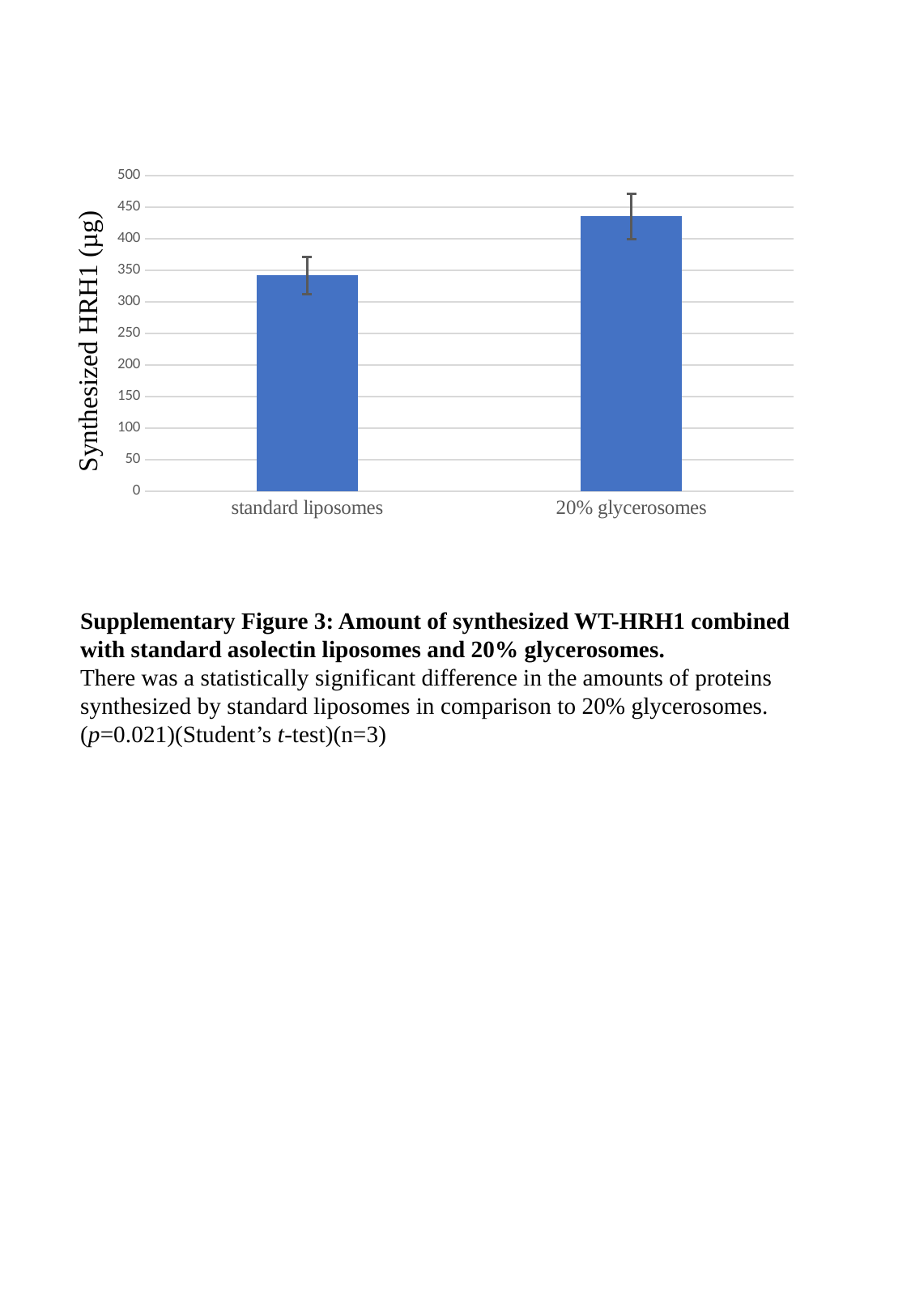

### Chart
| Category | |
|---|---|
| standard liposomes | 341.9 |
| 20% glycerosomes | 435.6 |Synthesized HRH1 (µg)
Supplementary Figure 3: Amount of synthesized WT-HRH1 combined with standard asolectin liposomes and 20% glycerosomes.
There was a statistically significant difference in the amounts of proteins synthesized by standard liposomes in comparison to 20% glycerosomes. (p=0.021)(Student’s t-test)(n=3)
