# Supplementary material for: Functional G-Protein-Coupled Receptor (GPCR) Synthesis: The Pharmacological Analysis of Human Histamine H1 Receptor (HRH1) Synthesized by a Wheat Germ Cell-Free Protein Synthesis System Combined with Asolectin Glycerosomes
Source: Front Pharmacol. 2018 Feb 6;9:38. doi: 10.3389/fphar.2018.00038 (PMC5808195; doi:10.3389/fphar.2018.00038)
Supplement: Supplementary file 4 [file Presentation_4.pptx]

## Slide 1
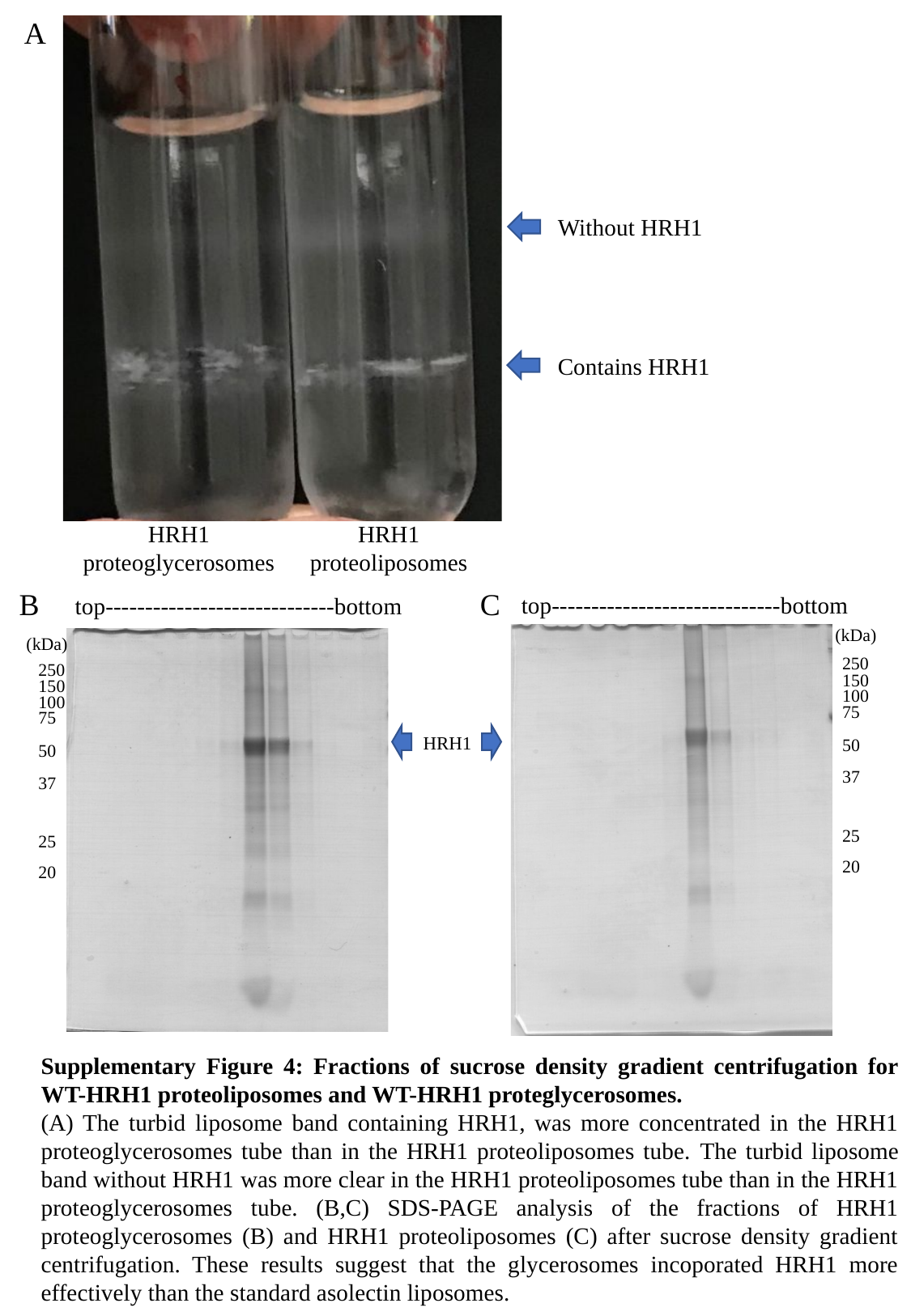

A
Without HRH1
Contains HRH1
HRH1 proteoglycerosomes
HRH1 proteoliposomes
C
B
top-----------------------------bottom
top-----------------------------bottom
(kDa)
(kDa)
250
250
150
150
100
100
75
75
HRH1
50
50
37
37
25
25
20
20
Supplementary Figure 4: Fractions of sucrose density gradient centrifugation for WT-HRH1 proteoliposomes and WT-HRH1 proteglycerosomes.
(A) The turbid liposome band containing HRH1, was more concentrated in the HRH1 proteoglycerosomes tube than in the HRH1 proteoliposomes tube. The turbid liposome band without HRH1 was more clear in the HRH1 proteoliposomes tube than in the HRH1 proteoglycerosomes tube. (B,C) SDS-PAGE analysis of the fractions of HRH1 proteoglycerosomes (B) and HRH1 proteoliposomes (C) after sucrose density gradient centrifugation. These results suggest that the glycerosomes incoporated HRH1 more effectively than the standard asolectin liposomes.
