# Supplementary material for: Functional G-Protein-Coupled Receptor (GPCR) Synthesis: The Pharmacological Analysis of Human Histamine H1 Receptor (HRH1) Synthesized by a Wheat Germ Cell-Free Protein Synthesis System Combined with Asolectin Glycerosomes
Source: Front Pharmacol. 2018 Feb 6;9:38. doi: 10.3389/fphar.2018.00038 (PMC5808195; doi:10.3389/fphar.2018.00038)
Supplement: Supplementary file 5 [file Presentation_5.pptx]

## Slide 1
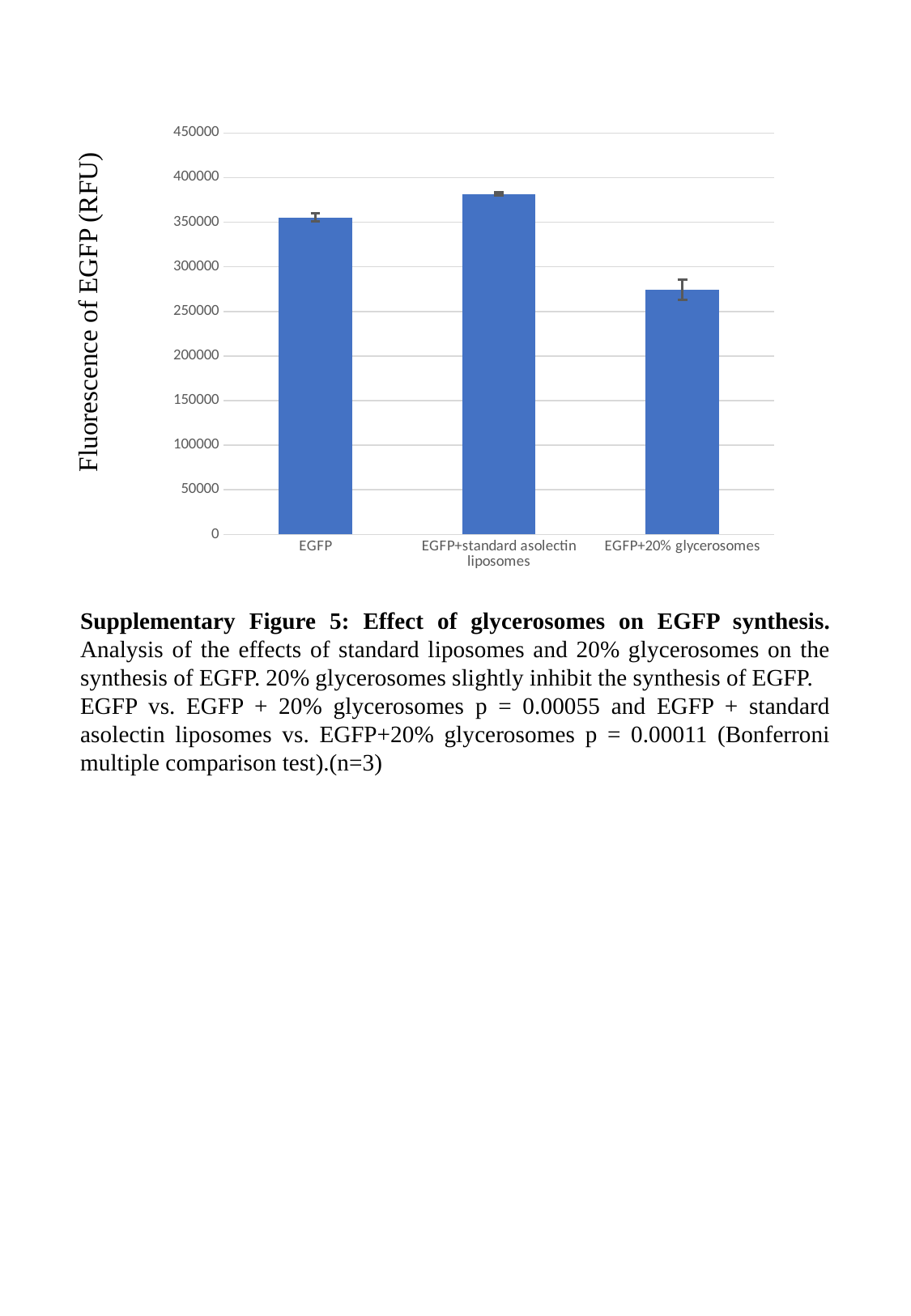

### Chart
| Category | fluorescence |
|---|---|
| EGFP | 355398.3333333333 |
| EGFP+standard asolectin liposomes | 381745.6666666667 |
| EGFP+20% glycerosomes | 274154.3333333333 |Fluorescence of EGFP (RFU)
Supplementary Figure 5: Effect of glycerosomes on EGFP synthesis. Analysis of the effects of standard liposomes and 20% glycerosomes on the synthesis of EGFP. 20% glycerosomes slightly inhibit the synthesis of EGFP.
EGFP vs. EGFP + 20% glycerosomes p = 0.00055 and EGFP + standard asolectin liposomes vs. EGFP+20% glycerosomes p = 0.00011 (Bonferroni multiple comparison test).(n=3)
